# Receipt of cancer care among stage IV non-small-cell lung cancer patients with and without intellectual or developmental disabilities: A population-based retrospective cohort study

**DOI:** 10.1177/02692163261464345

**Published:** 2026-07-23

**Authors:** Rebecca L. W. Hansford, Laura E. Davis, Rebecca Griffiths, Tara Horrill, Paul Nguyen, Catherine L. Goldie, Timothy P. Hanna, Alyson L. Mahar

**Affiliations:** 1 School of Nursing, Queen’s University, Kingston, ON, Canada; 2 Division of Cancer Care & Epidemiology, Sinclair Cancer Research Institute, Queen’s University, Kingston, ON, Canada; 3 ICES Queen’s, Queen’s University, Kingston, ON, Canada; 4 College of Nursing, University of Manitoba, Winnipeg, MB, Canada; 5 Department of Oncology, Queen’s University, Kingston, ON, Canada

**Keywords:** lung cancer, intellectual or developmental disabilities, cancer treatment, population-based retrospective cohort study, palliative care

## Abstract

**Background:**

Little is known about non-curative lung cancer care, including palliative care, for adults with intellectual, or developmental disabilities. Clinical management may vary due to health status, ability to communicate, access to inclusive healthcare, and healthcare bias.

**Aim:**

We examined the receipt of cancer-directed consultations, treatments and palliative care among stage IV non-small-cell lung cancer (NSCLC) patients with and without intellectual or developmental disabilities.

**Design:**

This was a population-based retrospective cohort study using provincial routinely collected health data. Receipt of consultations with surgeons, medical oncologists, and radiation oncologists, receipt of systemic therapy, radiation, and surgery as well as receipt of palliative care in the year following diagnosis were compared between people with and without intellectual or developmental disabilities. Cause-specific Cox proportional hazards regression, accounting for death as a competing event, was used.

**Setting/participants:**

Adults diagnosed with stage IV NSCLC between 2010 and 2022 in Ontario, Canada.

**Results:**

The study included 33,184 individuals diagnosed with stage IV NSCLC (n intellectual or developmental disabilities = 106). Adults with intellectual or developmental disabilities were significantly less likely to receive any cancer-directed consultation (hazard ratio [HR] = 0.62; 95% confidence interval [CI] 0.49–0.78), any cancer-directed cancer treatment (HR = 0.52; 95% CI 0.39–0.70), and radiation or systemic therapy (HR = 0.49; 95% CI 0.36–0.66) than non-disabled adults. There was no statistical difference in receipt of palliative care (HR = 1.15; 95% CI 0.93–1.41).

**Conclusion:**

These findings contribute to an emerging evidence base documenting differences in cancer treatment and outcomes among adults with intellectual or developmental disabilities. Person-center research is needed that examines end-of-life lung cancer care treatment decision-making to identify and mitigate barriers to optimal management.

**What is already known?**

- Adults with intellectual or developmental disabilities are more likely to die from lung cancer than non-disabled adults.
- There is limited research exploring palliative and end-of-life cancer care among adults with intellectual or developmental disabilities, including those with stage IV non-small-cell lung cancer.

**What this paper adds?**

- Cancer care, including rates of consultations and treatment rates, were lower among adults with intellectual or developmental disabilities compared to those without disabilities.
- Likelihood of palliative care was similar among adults with and without intellectual or developmental disabilities.

**Implications for practice, theory, or policy?**

- Research is needed to better understand treatment access and decision-making near the end-of-life among adults with intellectual or developmental disabilities with stage IV lung cancer.
- The development of neuroinclusive lung cancer programs could be beneficial in improving the accessibility of cancer care for adults with intellectual or developmental disability.

## Introduction

Intellectual or developmental disabilities refer to conditions that originate during the developmental period, which impact adaptive behavior, social skills, and intellectual functioning.^1^ Adults with intellectual or developmental disabilities are as likely to be diagnosed with metastatic lung disease^2^ and twice as likely to be unstaged at diagnosis.^3^ Research specific to lung cancer treatment, including receipt of palliative care, for adults with intellectual, or developmental disabilities does not exist. Adults with intellectual or developmental disabilities may experience worse treatment outcomes than those without disabilities, such as longer lengths of stay, and increased mortality following cancer surgical resection.^4^ These disparities may result from multiple factors, including more complex health diagnoses and difficulties with communication, in combination with health care systems that are not designed to support their needs.^5^ Additionally, people with intellectual or developmental disabilities are often excluded from randomized controlled trials,^6^ further decreasing the availability of information for treatment decision-making. Together, these may introduce opportunities for variation in clinical management. Therefore, this study compared receipt of cancer-directed consultations and treatment, including palliative care, between adults diagnosed with stage IV non-small-cell lung cancer (NSCLC) with and without intellectual or developmental disabilities to identify areas for future policy and research.

## Methods

### Design and setting

We conducted a population-based retrospective cohort study with routinely collected health administrative data held at ICES in Ontario, Canada. Ontario residents have access to universal, publicly funded healthcare insurance. The study received ethical clearance from the Queen’s Faculty of Health Sciences and Affiliated Hospitals Research Ethics Board (HSREB #NURS-559-22). ICES is an independent, non-profit research institute whose legal status under Ontario’s health information privacy law allows it to collect and analyze health care and demographic data, without consent, for health system evaluation and improvement. Datasets were linked using unique encoded identifiers and analyzed at ICES.

### Population

Ontarian adults (age 18+) diagnosed with stage IV NSCLC between January 2010, and September 2022 were included in the study and followed for up to 1 year from diagnosis. We included individuals from the Ontario Cancer Registry using a combination of ICD-O-3 morphology and topography codes (Table S1),^7^ who had Ontario Health Insurance Plan (OHIP) coverage on the date of diagnosis, had no history of a previous cancer diagnosis, and had provincial health coverage in the 2 years before their diagnosis.

### Identification of intellectual or developmental disabilities

Status of intellectual or developmental disabilities was identified using established algorithms based on the occurrence of healthcare encounters with associated ICD-9 and 10 codes (Table S2).^2^ An individual with ⩾1 eligible diagnoses in records from the emergency department records, home care, hospitalization, complex continuing care, or ⩾ 2 physician visits with eligible diagnoses at least 6 months before their cancer diagnosis was identified as having intellectual or developmental disabilities.

### Outcome definitions

Outcome definitions and data sources are provided in Table S1. Registration at an Regional Cancer Centre (RCC) was measured in patient-level regional cancer care records from 6 months before or after the cancer diagnosis. Cancer-directed consultations were defined as those visits occurring with a medical oncologist, radiation oncologist, or thoracic surgeon and identified using physician billing records from 60 days prior to diagnosis until death or the end of follow-up. Receipt of palliative care was defined broadly using physician billing codes in the 60 days prior to diagnosis and to death or end of follow-up, including but not limited to special care palliative care consultation and palliative care case management.^7^ Cancer-directed treatment included surgery (lobectomy, pneumonectomy, intracranial tumor resection, spinal cord compression surgery, liver excision), systemic therapy, and radiation. Treatment outcomes were measured using hospitalization discharges and intervention codes (surgery), physician billing codes (systemic therapy, surgery), and regional cancer center records (radiation) from the date of diagnosis to death or end of follow-up.^7^ Any treatment was defined as receipt of surgical resection, systemic therapy or radiation (yes/no). An additional aggregate outcome of any systemic therapy or radiation (yes/no) was considered.

### Data analysis

Baseline characteristics included age at diagnosis (years), sex (male, female), rurality (rural, suburban, urban), Elixhauser comorbidity score (0, 1–2, 3+),^8^ diagnosis year (calendar year), neighborhood income quintiles (1–5), and chronic conditions. Asthma, chronic obstructive pulmonary disorder (COPD), hypertension and congestive heart failure were identified using established ICES definitions.

Cause-specific Cox proportional hazards models were estimated to test the association between intellectual or developmental disabilities and receipt of any cancer-directed consultations, palliative care, and cancer-directed treatment accounting for death as a competing event.^9^ We included age, sex, year of diagnosis, and rurality as confounders based on an a priori conceptual model.^2,10^ We estimated additional models accounting for Elixhauser comorbidity score considering the critical role it plays in determining treatment selection. We conducted additional analyses using the Fine-Gray subdistribution hazard models.^11^ Data analyses were performed using the SAS software (version 9.4, SAS Institute, Cary, NC).

## Results

**Figure 1.**
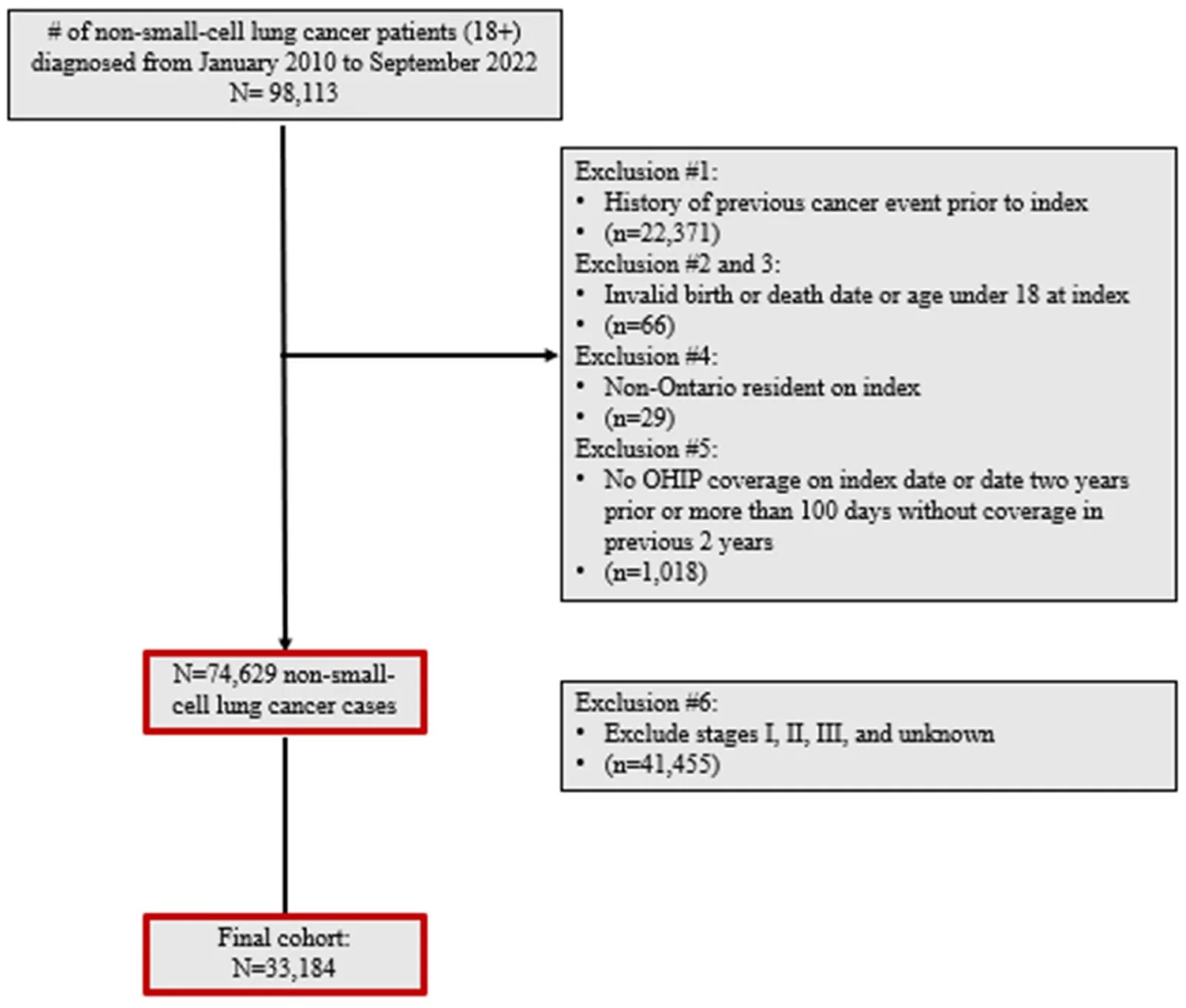
Cohort selection for lung cancer cohort.

There were 33,184 individuals with stage IV NSCLC included in the cohort (n with intellectual or developmental disabilities = 106; Figure 1). Table 1 summarizes cohort characteristics. Relative to non-disabled people, those with intellectual or developmental disabilities tended to be younger (*p* < 0.001) and reside in communities in the lowest income quintile (*p* = 0.009). A significantly larger proportion of adults with intellectual or developmental disabilities were diagnosed with asthma (27% vs 14%; *p* < 0.001) and COPD (66% vs 44%; *p* < 0.001) than non-disabled adults.

**Table 1.** Baseline characteristics across intellectual or developmental disabilities status in metastatic lung cancer patients.

Variable	Value	IDD (*N* = 106), *N* (%)	No IDD (*N* = 33,078), *N* (%)	*p*
Age group	18–64	59 (56)	10,395 (31)	<0.001
	65–69	18 (17)	5365 (16)	
	70–74	15 (14)	5528 (17)	
	75–79	7 (7)	4882 (15)	
	> 80	7 (7)	6908 (21)	
Sex at birth	Female	48 (45)	15,743 (48)	0.63
	Male	58 (55)	17,335 (52)	
Rurality	Urban	61 (58)	21,244 (64)	0.34
	Suburban	30 (28)	8107 (25)	
	Rural	15 (14)	3727 (11)	
Neighborhood income quintile	Lowest	42 (40)	8356 (25)	0.009
	2	23 (22)	7431 (22)	
	3	11 (10)	6297 (19)	
	4	16 (15)	5742 (17)	
	Highest	14 (13)	5145 (16)	
	Missing	0 (0)	107 (0.3)	
Elixhauser comorbidity score	0	33 (31)	14,425 (44)	0.002
	1–2	39 (37)	12,288 (37)	
	3+	34 (32)	6365 (19)	
Asthma	Yes	29 (27)	4652 (14)	<0.001
COPD	Yes	70 (66)	14,549 (44)	<0.001
Hypertension	Yes	57 (54)	20,175 (61)	0.13
CHF	Yes	15 (14)	3814 (12)	0.40
Year of diagnosis	2010–2013	29 (27)	12,125 (37)	0.0
	2014–2016	31 (29)	7712 (23)	
	2017–2019	22 (21)	7766 (23)	
	2020–2022	24 (23)	5475 (17)	

COPD: chronic obstructive pulmonary disorder; CHF: congestive heart failure; IDD: intellectual or developmental disabilities.

Adults with intellectual or developmental disabilities were significantly less likely to be registered at a regional cancer center than those without disabilities (63% vs 80%, *p* < 0.001). Compared to non-disabled adults, a smaller percentage of adults with intellectual or developmental disabilities received a cancer-related consult (70% vs 87%; *p* < 0.001) or received cancer-directed treatment (any treatment: 43% vs 65%, *p* < 0.001; systemic or radiation: 41% vs 64%; *p* < 0.001). Most people received at least one palliative care visit (86% vs 90%). After adjusting for potential confounding variables, adults with intellectual or developmental disabilities were significantly less likely to have a cancer-directed consultation with a medical oncologist, radiation oncologist, or thoracic surgeon (hazard ratio [HR]= 0.62; 95% confidence interval [CI] 0.49–0.78). Adults with intellectual or developmental disabilities were also less likely to receive any cancer-directed treatment (surgery, systemic, or radiation; HR = 0.52; 95% CI 0.39–0.70), and radiation and systemic therapy (HR = 0.49; 95% CI 0.36–0.66) compared to those without disabilities (Table 2). After adjustment, no differences in palliative care between those with and without intellectual or developmental disabilities were observed (HR = 1.15; 95% CI 0.93–1.41).

**Table 2.** The relationship between intellectual or developmental disabilities status and time to cancer-directed consultation, cancer-directed treatment, and palliative care within 1 year using multivariable Cox-proportional hazards models accounting for the competing risk of death.

Exposure	Cancer-directed consult		Palliative care		Cancer-directed treatment (systemic therapy, radiation or surgical)		Cancer-directed treatment (systemic therapy or radiation)	
IDD status	HR (95% CI)	aHR^†^ (95% CI)	HR (95% CI)	aHR^†^ (95% CI)	HR (95% CI)	aHR^†^ (95% CI)	HR (95% CI)	aHR^†^ (95% CI)
IDD *N* = 106	0.71 (0.57–0.90)	0.62 (0.49–0.78)	1.18 (0.96–1.44)	1.15 (0.93–1.41)	0.63 (0.47–0.84)	0.52 (0.39–0.70)	0.58 (0.43–0.78)	0.49 (0.36–0.66)
No IDD *N* = 33,078	1.00 (ref)		1.00 (ref)		1.00 (ref)		1.00 (ref)	

IDD: intellectual or developmental disabilities; HR: hazard ratio; aHR: adjusted hazard ratio; CI: confidence interval.

† Adjusted models controlled for age (continuous), sex (categorized), year of diagnosis (categorized), rurality (categorized), and Elixhauser comorbidity.

## Discussion

### Main findings

Adults with intellectual or developmental disabilities and stage IV NSCLC were less likely to receive cancer-directed consultations and treatment relative to those without disabilities. These findings align with previous studies indicating less intensive cancer treatment for patients with intellectual or developmental disabilities^12–14^ and add to the growing body of evidence documenting differences in how adults with intellectual or developmental disabilities are treated. Fewer consultations with medical oncologists and radiation oncologists may signal less access to the cancer system, where specialized palliative care service referrals and management may occur. Cancer-directed treatment may be less common among adults with intellectual or developmental disabilities due to communication challenges, and complications with decision-making, consent, and legal capacity.^5^ Structurally, cancer-directed treatment for adults with intellectual or developmental disabilities and advanced cancer may not be accessible.^5^ Further exploration of factors contributing to lower rates of cancer-directed consultation and treatment are needed, including improving our understanding of decision-making, preferences, and expectations among adults with intellectual or developmental disabilities with stage IV NSCLC as well as other types of advanced cancers. Research to identify and mitigate barriers to adapting metastatic NSCLC treatment delivery may be needed. Neuro-inclusive NSCLC programs may benefit health systems in achieving these goals.

Most adults with intellectual or developmental disabilities received at least one consultation with palliative care and had no difference in receipt of at least one palliative care consultation compared to non-disabled adults. This may mean that in this universal health care system access to palliative care among metastatic NSCLC patients is not impacted by disability status. However, if fewer adults with intellectual or developmental disabilities received cancer-directed treatment, we might anticipate a larger proportion of the population to receive a palliative care consultation. Research in cancer populations with intellectual or developmental disabilities has documented fewer healthcare specialist visits at end of life, less routine symptom assessment and higher symptom scores, and fewer prescriptions for pain medication for adults with intellectual or developmental disabilities.^15–18^ Together with our findings of similar but not higher rates of palliative care consultations in the context of less cancer-direct care, these suggest further exploration into the extent and quality of palliative care delivered to metastatic cancer patients with intellectual or developmental disabilities is needed.

Our study did not explore how many consultations occurred, with which providers, the types of supports and services offered and accepted, nor whether the interactions were inclusive or person-centered and in alignment with the wishes of the adults with intellectual or developmental disabilities. Previous research has identified barriers to palliative care for adults with intellectual or developmental disabilities, including limited staff knowledge training and education, challenges with staff in assessing needs of persons with communication differences, and issues with care delivery, such as limited staffing, funding, and management support.^19^ Although palliative care guidelines specific for supporting adults with intellectual or developmental disability have been established, none are specific to the cancer context.^5,13^

### Strengths and limitations

This initial exploration of stage IV lung cancer-directed consultation and treatment as well as palliative care consultation among adults with intellectual or developmental disabilities is limited by our algorithm that cannot differentiate intellectual or developmental disability conditions or disability profoundness. Rates of cancer-directed consultations, treatment and palliative care could be lower or higher among subpopulations of adults with intellectual or developmental disabilities and lung cancer. This first description may overestimate the intensity of cancer-directed treatment and palliative care among adults with intellectual or developmental disabilities if they are less likely to have follow-up visits or complete recommended treatments in full than those without disabilities.

## Conclusion

Research to identify and mitigate barriers to adapting palliative lung cancer care delivery at end-of-life may be needed, including the development of cancer-specific palliative care guidelines for supporting cancer patients with intellectual or developmental disabilities.^5,13^ Differences in cancer-directed treatment and symptom management may explain the worse lung cancer survival observed among adults with intellectual or developmental disabilities.^20,21^ More research is needed to explore lung cancer treatment decision-making and end-of-life care and treatment preferences for adults with intellectual or developmental disabilities to ensure opportunities to provide person-centered, inclusive lung cancer treatment and palliative care.

## Supplemental Material

sj-docx-1-pmj-10.1177_02692163261464345 – Supplemental material for Receipt of cancer care among stage IV non-small-cell lung cancer patients with and without intellectual or developmental disabilities: A population-based retrospective cohort study Supplemental material, sj-docx-1-pmj-10.1177_02692163261464345 for Receipt of cancer care among stage IV non-small-cell lung cancer patients with and without intellectual or developmental disabilities: A population-based retrospective cohort study by Rebecca L. W. Hansford, Laura E. Davis, Rebecca Griffiths, Tara Horrill, Paul Nguyen, Catherine L. Goldie, Timothy P. Hanna and Alyson L. Mahar in Palliative Medicine

## Data Availability

The dataset from this study is held securely in coded form at ICES. While legal data sharing agreements between ICES and data providers (e.g. healthcare organizations and government) prohibit ICES from making the dataset publicly available, access may be granted to those who meet pre-specified criteria for confidential access, available at www.ices.on.ca/DAS (email: das@ices.on.ca). The full dataset creation plan and underlying analytic code are available from the authors upon request, understanding that the computer programs may rely upon coding templates or macros that are unique to ICES and are therefore either inaccessible or may require modification.
