# Supplementary material for: Receipt of cancer care among stage IV non-small-cell lung cancer patients with and without intellectual or developmental disabilities: A population-based retrospective cohort study
Source: Palliat Med. 2026 Jul 23;40(9):1400–7. doi: 10.1177/02692163261464345 (PMC13612864; doi:10.1177/02692163261464345)
Supplement: Supplementary file 1 [file sj-docx-1-pmj-10.1177_02692163261464345.docx]

Supplemental Table 1. Definitions for identifying non-small-cell lung cancer and outcome variables.

| **Variable name** | **Data source** | **Code strategy** |
| --- | --- | --- |
| **Non-small cell lung cancer** | OCR | Presence of both morphology and topography for NLSC:  Morphology codes: 80003, 80013, 80043, 80103, 80123, 80133, 80203, 80213, 80223, 80303, 80313, 80323, 80343, 80463, 80503, 80513, 80523, 80703, 80706, 80713, 80716, 80723, 80733, 80743, 80753, 80763, 80823, 80833, 80843, 80943, 81203, 81303, 81403, 81406, 81413, 81433, 81443, 81453, 81903, 82003, 82013, 82103, 82113, 82303, 82313, 82503, 82513, 82523, 82533, 82543, 82553, 82603, 82613, 82633, 82653, 82903, 83103, 83233, 83303, 83333, 83403, 83803, 83843, 84013, 84103, 84203, 84303, 84403, 84413, 84423, 84623, 84703, 84723, 84803, 84813, 84903, 84906, 85003, 85073, 85103, 85503, 85603, 85623, 85703, 85723, 85743, 85753, 88023, 89803  Topography codes: C340, C341, C342, C343, C348, C349 |
| **Palliative care treatment** | OHIP | Any of the following from 60 days prior to diagnosis to death or end of follow up: A901 (only if billed with B997 or B998), A902 (only if billed with B997 or B998), A945, C882, C945, C982, G511, G512, K015, K023, K700, W872, W882, W982 |
| **Cancer-directed consultation** | | |
| **Medical oncology** | OHIP | Any of the following in the 60 days prior to diagnosis to death or the end of follow up:  A445, A135, A615, A130, A446, A435, A655, A845, A136, A616, A443, A133, A613, A441, A131, A611, A444, A134, A614, A448, A138, A618, C445, C845, C446, C443, C444, C441, W445, W845, W446, A135, A130, A435, A136, A133, A131, A134, A138, C135, C130, C435, C136, C133, C134, C131, W234, W130, W435, W236, A615, A655, A616, A613, A611, A614, A618, C615, C655, C616, C613, C614, C611  Must be billed by a physician that also billed ≥2 of: G381, G281, G339, G345, G359 |
| **Radiation oncology** |  | Any of the following in the 60 days prior to diagnosis to death or the end of follow up:  Radiation oncology consultation, OHIP: A345, A745, A346, C345, C745, C346 |
| **Thoracic surgery** |  | Any of the following in the 60 days prior to diagnosis to death or the end of follow up:  Thoracic surgery consultation, OHIP: A645, A935, A646, A643, A644, C645, C935, C646, C643, W645, W646 |
| **Cancer-directed treatment** | | |
| **Systemic therapy** | OHIP | Receipt of any of the following from date of diagnosis to death or end of follow up:  Systemic therapy: OHIP fee codes G381, G281, G339, G345, G359 |
| **Radiation** | ALR | Receipt of any of the following from date of diagnosis to death or end of follow up:  Presence of any ALR NHPIP codes: 500, 501, 503, 510, 511, 512, 519, 520, 521, 522, 523, 524, 525, 526, 527, 528, 529, 530, 531, 532, 533, 534, 535, 536, 537, 538, 539, 540, 541, 542, 543, 544, 548, 549, 561, 563, 565, 566, 568, 570, 571, 572, 573, 574, 575, 576, 577, 581, 582, 590, 591, 592, 593, 594, 596, 597, 598, 599 |
| **Surgical** | DAD | Receipt of any of the following from date of diagnosis to death or end of follow up:  Lobectomy or pneumonectomy, DAD CCI codes: 1GR87, 1GR89, 1GR91, 1GT59, 1GT87, 1GT89, 1GT91, 1GM87, 1GW59, 1GW87, 1GX87  Intracranial tumour resection, DAD CCI codes: 1AA87, 1AB87, 1AC87, 1AE87, 1AG87, 1AJ87, 1AK87, 1AN59, 1AN87, 1AP87  Spinal cord compression, DAD CCI codes: 1AW72, 1AX87, 1SC80, 1SC87, 1SC89  Liver resection, DAD CCI codes: 1OA87  Thoracic tumour resection, DAD CCI codes: 1GM87, 1GR87, 1GR89, 1GR91, 1GT59, 1GT87, 1GT89, 1GT91, 1GW59, 1GW87, 1GX87 |

Supplemental Table 2. Algorithm for identifying IDD status.

| **Diagnostic Code** | **Description** |
| --- | --- |
| **ICD-9** | |
| 299-2999 | Pervasive development disorders (e.g., autism) |
| 317-3179 | Mental retardation |
| 318-3189 | Mental retardation |
| 319-3199 | Mental retardation |
| 7580-7583 | Chromosomal anomalies for which a developmental disability is typically present |
| 7585 | Other conditions due to autosomal anomalies |
| 7588 | Other conditions due to chromosome anomalies |
| 7589 | Conditions due to anomaly of unspecified chromosome |
| 7595 | Tuberous sclerosis |
| 75981 | Other and unspecified congenital anomalies: Prader Willi |
| 75982 | Marfan Syndrome |
| 75983 | Other and unspecified congenital anomalies: fragile x |
| 75989 | Other and unspecified congenital anomalies: other (E.g. menkes disease, Laurence-Moon-Biedl,rubinstein-taybi syndrome etc.) |
| 76070 | NOXIOUS SUBST NOS AFF NB |
| 76071 | NOXIOUS SUBST NOS AFF NB |
| 76072 | MATERNAL ALCOHOL AFF NB |
| 76072 | MATERNAL NARCOTIC AFF NB |
| 76073 | MATERNAL HALLUCIN AFF NB |
| 76074 | MATERNAL ANTI-INF AFF NB |
| 76075 | COCAIN AFF FET VIA PLAC/BR MILK |
| 76077 | Fetal hydantoin syndrome |
| 76079 | NOXIOUS SUBST NEC AFF NB |
| **ICD-10** | |
| F70/F700 | Mild mental retardation with the statement of no, or minimal, impairment of behaviour |
| F701 | Mild mental retardation, significant impairment of behaviour requiring attention or treatment |
| F708 | Mild mental retardation, other impairments of behaviour |
| F709 | Mild mental retardation without mention of impairment of behaviour |
| F71/F710 | Moderate mental retardation with the statement of no, or minimal, impairment of behaviour |
| F711 | Moderate mental retardation, significant impairment of behaviour requiring attention or treatment |
| F718 | Moderate mental retardation, other impairments of behaviour |
| F719 | Moderate mental retardation without mention of impairment of behaviour |
| F72/F720 | Severe mental retardation with the statement of no, or minimal, impairment of behaviour |
| F721 | Severe mental retardation, significant impairment of behaviour requiring attention or treatment |
| F728 | Severe mental retardation, other impairments of behaviour |
| F729 | Severe mental retardation without mention of impairment of behaviour |
| F73/F730 | Profound mental retardation with the statement of no, or minimal, impairment of behaviour |
| F731 | Profound mental retardation, significant impairment of behaviour requiring attention or treatment |
| F738 | Profound mental retardation, other impairments of behaviour |
| F739 | Profound mental retardation without mention of impairment of behaviour |
| F78/F780 | Other mental retardation with the statement of no, or minimal, impairment of behaviour |
| F781 | Other mental retardation, significant impairment of behaviour requiring attention or treatment |
| F788 | Other mental retardation, other impairments of behaviour |
| F789 | Other mental retardation without mention of impairment of behaviour |
| F79/F790 | Unspecified mental retardation with the statement of no, or minimal, impairment of behaviour |
| F791 | Unspecified mental retardation, significant impairment of behaviour requiring attention or treatment |
| F798 | Unspecified mental retardation, other impairments of behaviour |
| F799 | Unspecified mental retardation without mention of impairment of behaviour |
| F840 | Childhood autism |
| F841 | Atypical autism |
| F842 | Rett’s Syndrome |
| F844 | Overactive disorder associated with mental retardation and stereotyped movements |
| F845 | Asperger’s syndrome |
| F848 | Other pervasive developmental disorders |
| F849 | Pervasive developmental disorder, unspecified |
| Q851 | Tuberous sclerosis |
| Q860 | Fetal Alcohol Syndrome |
| Q861 | Fetal hydantoin syndrome |
| Q871 | Aarskog, Prader willi, DeLange, Seckel etc. |
| Q878 | Other |
| Q900-Q939 **except** Q926 | (i.e., all Down Syndrome Types, cri du chat, etc **except** Extra marker chromosomes) |
| Q971 | Female with more than three X chromosomes |
| Q992 | Fragile X |
| Q998 | Other specified chromosome abnormalities |

Supplemental Table 3. The relationship between IDD status and time to cancer-directed consultation, cancer-directed treatment, and palliative care within 1 year using Fine and Gray subdistribution hazards models.

| **Exposure** | **Cancer-directed consult** | | **Palliative care** | | **Cancer-directed treatment**  **(Systemic therapy, radiation or surgical)** | | **Cancer-directed treatment (Systemic therapy or radiation)** | |
| --- | --- | --- | --- | --- | --- | --- | --- | --- |
| IDD status | HR  (95% CI) | aHR  (95% CI) | HR  (95% CI) | aHR  (95% CI) | HR  (95% CI) | aHR  (95% CI) | HR  (95% CI) | aHR  (95% CI) |
| IDD  N=106 | 0.65  (0.50- 0.84) | 0.58  (0.46- 0.74) | 1.08  (0.85- 1.38) | 1.07  (0.84- 1.36) | 0.56  (0.42- 0.76) | 0.48  (0.36- 0.65) | 0.52  (0.38- 0.70) | 0.45  (0.34- 0.61) |
| No IDD N=33,078 | 1.00 (ref) | | 1.00 (ref) | | 1.00 (ref) | | 1.00 (ref) | |

^†^Adjusted models controlled for age (continuous), sex (categorized), year of diagnosis (categorized), rurality (categorized), and Elixhauser comorbidity.

IDD= intellectual or developmental disabilities

HR= hazard ratio

aHR= adjusted hazard ratio

CI= confidence interval
